# Synthesis and Structure of Sulfur Derivatives from 2-Aminobenzimidazole

**DOI:** 10.3390/molecules190913878

**Published:** 2014-09-04

**Authors:** Alejandro Cruz, Itzia I. Padilla-Martínez, Efrén V. García-Báez, Gerardo Guerrero-Muñoz

**Affiliations:** Instituto Politécnico Nacional-UPIBI, Departamento de Ciencias Básicas, Av. Acueducto s/n Barrio la Laguna Ticomán, 07340 México, D.F., Mexico; E-Mails: ipadillamar@ipn.mx (I.I.P.-M.); efren1003@yahoo.com.mx (E.V.G.-B.); ger_2202@hotmail.com (G.G.-M.)

**Keywords:** 2-aminobenzimidazole, dithiocarbamates, *S*-methyldithiocarbamates, dithio-methylcarboimidates

## Abstract

The reactions of the benzimidazole nitrogen atoms and the exocyclic amino group of 2-aminobenzimidazole with CS_2_ in NaOH basic medium followed by methylation with methyl iodide was explored. With careful control of the stoichiometric quantities and addition sequences, this set of reactions allows the selective functionalization of the benzimidazole ring with *N*-dithiocarbamate, *S*-methyldithiocarbamate or dimethyl- dithiocarboimidate groups. The products were characterized by ^1^H-, ^13^C-NMR spectroscopy and three of them by X-ray diffraction analysis. The preferred isomers, tautomers and conformers were established.

## 1. Introduction

We are currently investigating the structures of biologically active benzofused nitrogen heterocycles such as 2-aminobenzazoles [1,2,3,4]. They are versatile from the structural point of view because of their free lone pairs, labile hydrogen atoms, and planar delocalized acyclic groups. The delocalized 10-π electronic system and the extended electronic conjugation with the amino group, make these heterocycles have amphoteric character. Moreover, 2-aminobenzazoles [5,6] occur in broad spectrum of drugs and pharmacological agents with anticancer, antibacterial, antiviral, analgesic, antidiabetic properties. Thus, several 2-aminobenzimidazole-derived drugs are registered around the world. For example, mebendazole represents a big group of antiparasitic drugs, and astemizole represents an antihistaminic group II generation drug with selective activity toward H1 receptors.

**Scheme 1:**
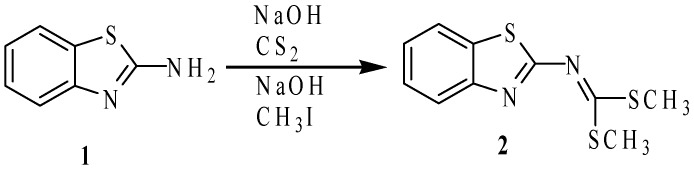
Synthesis of dimethyl benzo[*d*]thiazol-2-carbonodithioimidate (**2**).

In this context, we have reported a detailed study and characterization of the intermediates involved in the synthesis of dimethyl benzo[*d*]thiazol-2-carbonodithioimidate (**2**) [7], by the reaction of 2-aminobenzothiazole (**1**) with carbon disulfide in basic media, following the procedure reported by Merchand *et al.* [8] (Scheme 1). Compound **2** reacts with *ortho*-XH substituted anilines in refluxing DMF to give NH-bisbenzazoles [9,10,11] due to the facility with which HSMe molecules are displaced. We used this method to prepare chiral 2-iminobenzothiazole heterocycles derived from ephedrine [12]. On this basis, we reported a series of sulfur compounds such as thiourea, isothiourea, dithiocarbamate, dithiocarboimine, dimethyldithiocarbamate, methyldithiocarbamate, S-methyl and O-alkyl thiocarbamic esters derived from 2-aminobenzothiazole [7]. These new compounds are versatile because they have very reactive functional groups, and thus they can be used as intermediates for the synthesis of more complex molecules. Besides, they possess rigid frameworks and several lone pairs available for coordination and then they are potentially interesting ligands for metallic coordinating compounds. On the other hand, we have also recently reported a synthetic method to access symmetric and non-symmetric 2-(*N*,*N'*-disubstituted)-guanidinebenzothiazoles [13] from the reaction of ammonia, methylamine, pyrrolidine and aniline with compound **2**, isolating isothioureas as intermediates [14]. 

The presence of amine groups of different orders in 2-aminobenzimidazole enables the synthesis of various structural derivatives. In this case, we are interested in the functionalization of amino groups in 2-aminobenzimidazole (**3**) with sodium hydroxide, carbon disulfide and methyl iodide to give sulfur derivatives.

## 2. Results and Discussion

### 2.1. Synthesis

**Scheme 2:**
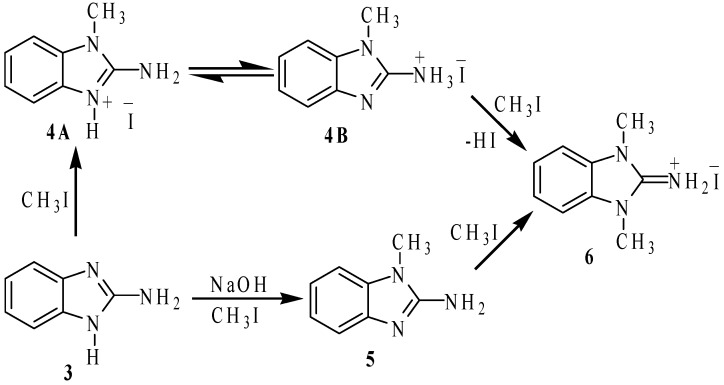
Methylation reactions of 2-aminobenzimidazole (**3**).

To investigate the nitrogen nucleophilicity, we reacted 2-aminobenzimidazole with methyl iodide (MeI) in ethanol as solvent (Scheme 2). If one molar equivalent of MeI was added, a mixture of the iodide salts: 1-methyl-2-ammoniumbenzimidazole (**4**, 30%), 1,3-dimethyl-2-ammoniumbenzimidazole (**6**, 30%) and 2-ammoniobenzimidazole (30%) were observed in the ^1^H-NMR spectrum. To explain this result, we propose that 2-aminobenzimidazole is methylated to produce the iodide salt of the *N*-methylated compound **4A** as intermediate, which is immediately transformed into the more stable tautomer **4B**. When two molar equivalents of methyl iodide were used, the basic imidazolic nitrogen atom of the iodide salt **4B** is methylated to give the dimethylated iodide salt **6** (60%). The remaining 2-aminobenzimidazole traps the generated HI to give the iodide salt of 2-ammoniumbenzimidazole (30%), as side product.

In order to isolate the *N*-methylbenzimidazole **5**, we reacted 2-aminobenzimidazole (**3**) with one molar equivalent of sodium hydroxide using as DMF solvent, followed by one molar equivalent of MeI, as depicted in Scheme 2. Under these conditions, the reaction afforded a 1:1:1 mixture of **5**, the dimethylated iodide salt **6** and unreacted **3**. The same reaction with two molar equivalents of MeI affords **6** as the only product in 90% yield. 

**Scheme 3:**
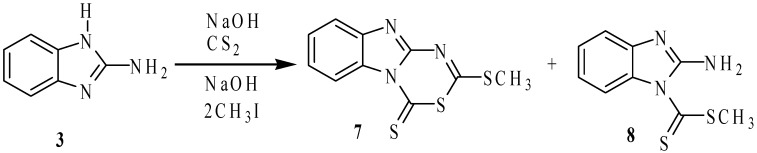
Reaction of 2-aminobenzimidazole (**3**) with CS_2_ and methyl iodide in basic media.

On these bases, a detailed study and the characterization of the compounds derived from the reaction of 2-aminobenzimidazole (**3**) with carbon disulfide in basic media followed by methylation with methyl iodide, using DMF as solvent were performed. When **3** was reacted with CS_2_ and CH_3_I in basic (NaOH) medium, following the reported procedure for the synthesis of compound **2** [8], the reaction failed to give the analogous compound, and instead a 1:3 mixture of compounds **7** and **8** were observed in the ^1^H- and ^13^C-NMR spectra (Scheme 3). With separation purposes, this mixture was dissolved in ethanol. 2-Methylthio-4*H*-[1,3,5]thiadiazino[3,4-*a*]benzimidazole-4-thione (**7**) was isolated from the mixture as an insoluble yellow solid and after recrystallization from chloroform, it was obtained as yellow crystals in 15% yield. 2-Aminobenzimidazole-1-carbodithioic acid methyl ester (**8**) precipitated from the ethanol solution, as yellow crystals in 40% yield as the previously described polymorph [15]. In this reaction, the small quantity of compound **7** is formed from **8** because CH_3_I, NaOH and CS_2_ were added in 20% molar excess. Compounds **7** and **8** were obtained in 40% and 66% yield, respectively, when the reactions were carried out in stoichiometric quantities. These results are in agreement with the stronger acidic character of the imidazolic hydrogen atom than that of the exocyclic 2-amino group. 

**Scheme 4:**
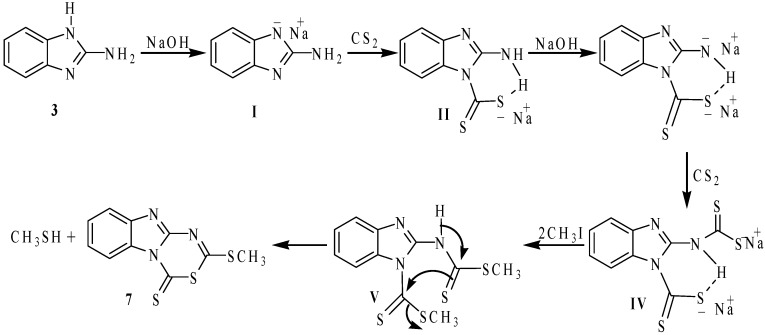
Mechanistic pathway to obtain compound **7**.

The formation of compounds **7** and **8** can be explained by assuming that the benzimidazolate **I**, obtained from the reaction of **3** with NaOH, which reacts with one molar equivalent of CS_2_ to give the thiocarbamate **II** that, after methylation, affords compound **8**. The intermediate **III**, formed by a second molar equivalent of NaOH, reacts with a second molar equivalent of CS_2_ to produce the dithiocarbamate dianion **IV**. The subsequent methylation of **IV** with two molar equivalents of MeI, affords the proposed intermediate compound **V**, which is finally cyclized to give the heterocyclic compound **7**. This mechanistic proposal is depicted in Scheme 4.

**Scheme 5:**
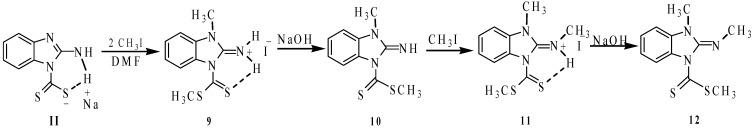
Synthetic method to get compounds **9**–**12**.

In order to obtain the compound **10**, we reacted 2-aminobenzimidazole (**3**) with exactly one molar equivalent of NaOH and one molar equivalent of CS_2_ followed by the addition of two molar equivalents of MeI. Under these conditions, the iminium iodide salt **9** was formed. Compound **9** was neutralized with NaOH to give 2-imino-3-methyl-2,3-dihydro-benzimidazol-1-carbodithioic acid methyl ester (**10**). Subsequent methylation at the imine nitrogen of compound **10** was performed to give *N*-(1-methyl-3-(methylthiocarbonothioyl)-1*H*-benzo[*d*]imidazol-2(3*H*)-ylidene)methanaminium iodide (**11**). 3-Methyl-2-methylimino-2,3-dihydro-benzoimidazole-1-carbodithioic acid methyl ester (**12**) was obtained by deprotonating **11** with NaOH. The sequence of reactions is depicted in Scheme 5. 

**Scheme 6:**
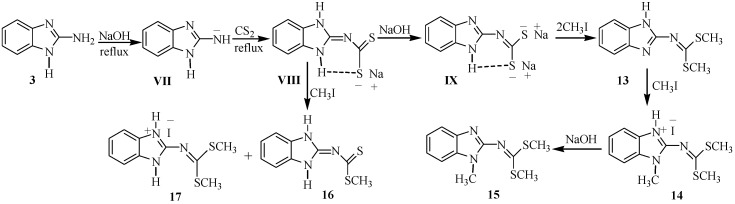
Synthetic pathway to access to compounds **13**–**17**.

The reaction of 2-aminobenzimidazole with NaOH and CS_2_ in an equimolar ratio, in refluxing DMF by 8 h was carried out, then one molar equivalent of NaOH and two molar equivalents of CH_3_I were subsequently added. Under these conditions, a white solid precipitates from the aqueous-DMF solution. The solid compound was purified by recrystallization in ethanol and white crystals were obtained in 25% yield. This compound was characterized by NMR and X-ray diffraction analysis and the structure corresponded to dimethyl 1*H*-benzo[*d*]imidazol-2-ylcarbonodithioimidate (**13**, Scheme 6). The reaction proceeded through the intermediacy of the exocyclic sodium amide **VII** whose formation is favored by heating. 

When three molar equivalents of methyl iodide were used in the methylation reaction to obtain **13**, the hydroiodide salt of dimethyl 1-methyl-1*H*-benzo[*d*]imidazol-2-ylcarbonodithioimidate (**14**) precipitated from the aqueous-DMF solution and was crystallized from ethanol. The structure of this compound was analyzed by X-ray diffraction (*vide infra*). After neutralization of **14** with one equivalent of NaOH, dimethyl 1-methyl-1*H*-benzo[*d*]imidazol-2-ylcarbonodithioimidate (**15**) was obtained quantitatively as a white solid. This compound has already been obtained from 1-methyl-2-aminobenzimidazole whose NMR data and X-ray diffraction structure has been reported elsewhere [16]. 

In order to obtain (1,3-dihydrobenzoimidazol-2-ylidene)-dithiocarbamic acid methyl ester (**16**), the intermediate **VIII** was prepared *in situ* and then methylated with one molar equivalent of CH_3_I, to afford compound **16** in mixture with the iodide salt of 2-(*bis*(methylthio)methyleneamino)-1*H*-benzo[*d*]imidazol-3-ium (**17**) which was separated as a precipitate from ethanol solution. 

### 2.2. Molecular Structure in Solution by NMR

A complete list of ^1^H and ^13^C-NMR data of compounds **7** and **9**–**14** and **16**–**17** is given in Table 1 and Table 2, respectively, to support the proposed structures. 

**Table 1:** ^1^H-NMR chemical shifts of compounds **7**, **9**–**14**, **16** and **17**. 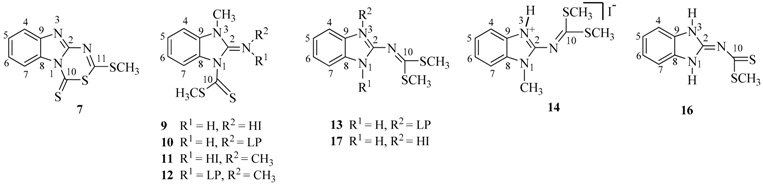

Comp.	H4	H5	H6	H7	NH	NCH_3_	SCH_3_
**7** ^b^	7.82	7.43	7.54	9.00	–	–	2.80
**9** ^a^	7.58	7.46	7.31	7.24	9.3	3.67	2.91
**10** ^b^	6.85	7.19	6.98	8.10	7.3	3.41	2.78
**11** ^a^	7.59	7.41	7.31	7.61	9.1	3.71, 3.10	2.96
**12** ^b^	6.77	6.93	7.11	8.12	–	3.40, 3.25	2.71
**13** ^b^	7.50	7.10	7.11	7.32	12.2	–	2.57
**14** ^a^	7.78	7.47	7.45	7.60	8.6	3.77	2.73
**16**	7.24	7.49	7.49	7.24	13.0	–	2.44
**17**	7.10	7.40	7.40	7.10	–	–	2.55

^a^ DMSO-*d*_6_; ^b^ CDCl_3_, LP = Lone pair.

**Table 2:** ^13^C-NMR chemical shifts of compounds **7**, **9**–**14**, **16** and **17**.

Comp.	C2	C4	C5	C6	C7	C8	C9	C10	C11	NCH_3_	SCH_3_
**7** ^a^	172.3	127.9	120.4	125.8	118.0	131.8	142.4	182.8	172.4	–	14.3
**9** ^b^	148.6	125.6	111.9	124.8	111.6	129.4	131.0	200.3	–	30.6	22.8
**10** ^a^	151.5	125.9	122.3	121.4	118.9	129.1	132.7	201.6	–	28.5	20.4
**11** ^a^	149.0	125.7	124.8	111.3	111.3	129.7	131.1	201.4	–	31.6, 31.3	23.0
**12** ^b^	145.5	124.4	120.1	112.5	106.3	131.0	134.5	203.3	–	36.0, 29.8	21.5
**13** ^a^	153.2	118.9	121.8	118.9	111.1	133.0	143.0	173.5	–	–	16.1
**14** ^a^	149.2	114.7	125.3	125.8	114.7	130.8	132.1	185.8	–	30.7	16.7
**16** ^b^	151.2	112.8	124.2	124.2	112.8	129.1	129.1	205.6	–	–	18.6
**17** ^a^	152.9	114.8	121.8	121.8	114.8	138.0	138.0	173.4	–	–	15.9

^a^ DMSO-*d*_6_; ^b^ CDCl_3_.

**Figure 1:**
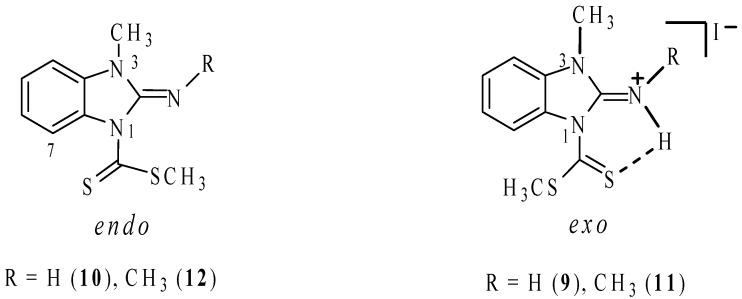
*Endo* conformers of compounds **10** and **12** and *exo* ones of compounds **9** and **11**.

**Scheme 7:**
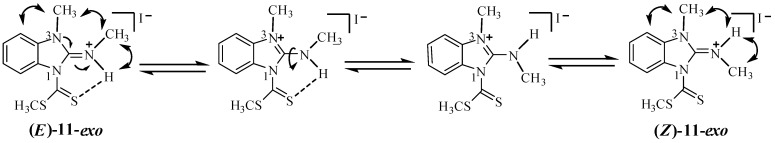
Isomerization of **11**-*exo* in DMSO-*d*_6_ solution. nOes are represented by double headed curved arrows.

The chemical shift of H7 in compounds **7** and **9**–**12**, is sensitive to the conformation of the C=S group. It appears as a doublet at δ 9.0 in compound **7** because of the deshielding effect of the neighbouring thiocarbonyl group. In this context, it is worth noting that the chemical shift of H7 for the neutral compounds **10** and **12**, is approximately at 8.1; this shift to higher frequencies suggests that the preferred conformation of the thiocarbonyl group on the NMR time scale is *endo* (Figure 1). In contrast, the hydrogen atom on the exocyclic nitrogen atom in imminium compounds **9** and **11**, forms a hydrogen bond with the sulfur atom of the thiocarbonyl group. This interaction has the effect to fix the conformation of the thiocarbonyl group in *exo* disposition, as has been reported for compound **8**, [15] shifting H7 to lower frequencies at 7.24 and 7.61 ppm, respectively (Scheme 7).

**Figure 2:**
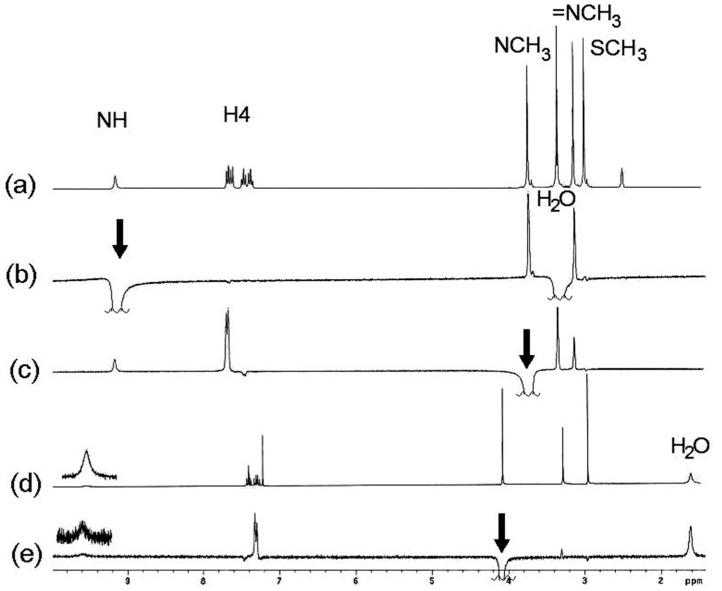
(**a**) ^1^H-NMR spectrum of compound **11** in DMSO-*d*_6_; (**b**) After irradiation of the NH signal, nOes on both CH_3_ groups are observed; (**c**) nOe on the NH, exocyclic NCH_3_ and H4 after irradiation of the N3-CH_3_ signal; (**d**) ^1^H-NMR spectrum of compound **11** in CDCl_3_; (**e**) Irradiation of N3-CH_3_ gave a nOe on NCH_3_ in CDCl_3_.

In order to confirm the stereochemistry of compound **11**, nOe experiments were carried out. Irradiation of the NH gave a nOe on both N-CH_3_ groups (Figure 2b) and the irradiation of the N3-CH_3_ protons gave a nOe on both the CH_3_ and NH protons of the exocylic C=N group (Figure 2c), in DMSO-*d*_6_ solution. After the irradiation of the N3-CH_3_ signal, nOe was observed only on the NH signal in CDCl_3_ solution (Figure 2e). In every case, the nOe was not observed on the SCH_3_ signal. These findings can be explained due to an isomerization process mediated by the participation of the lone pair of electrons on N3 (Scheme 7). In DMSO-*d*_6_ solution both *E* and *Z* isomers are in a fast equilibrium in the ^1^H-NMR time scale, whereas in CDCl_3_ solution the last isomer is the preferred.

**Figure 3:**
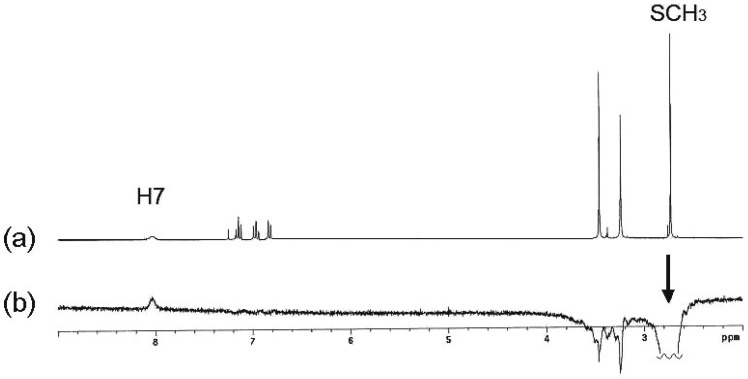
(**a**) ^1^H-NMR spectrum of compound **12** in CDCl_3_; (**b**) After irradiation of the SCH_3_ signal, nOe on H7 was observed.

**Scheme 8:**
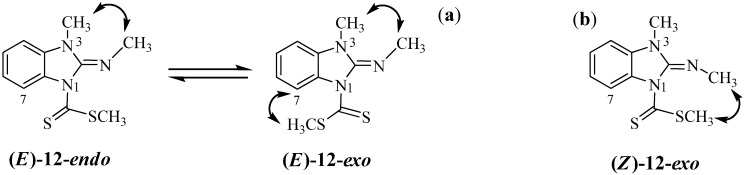
*Endo-exo* equilibrium in compound (***E***)-**12**. Expected nOes are represented by double headed curved arrows in *E* (**a**) and *Z* (**b**) isomers of compound **12**.

The shift of this equilibrium to the (***E***)-**11**-*exo* isomer in polar solvents, explains the isolation of the neutral compound **12** with both NCH_3_ groups in *syn* disposition and preference for the *endo* rotamer, on the ^1^H-NMR time scale. The stereochemistry of compound (***E***)**-12** was confirmed by nOe experiments (Figure 3). After irradiation of the SCH_3_ signal, both in DMSO-*d*_6_ and CDCl_3_ solutions, a very small nOe was observed on the H7 proton but not on the exocyclic NCH_3_, as expected for the (***Z***)**-12-***exo* isomer (Scheme 8). nOe on N3-CH_3_ was useless because the close proximity with the chemical shift of the exocyclic N-CH_3_.

### 2.3. Molecular Structure of Compounds ***7***, ***13*** and ***14*** by X-Ray Diffraction

**Figure 4:**
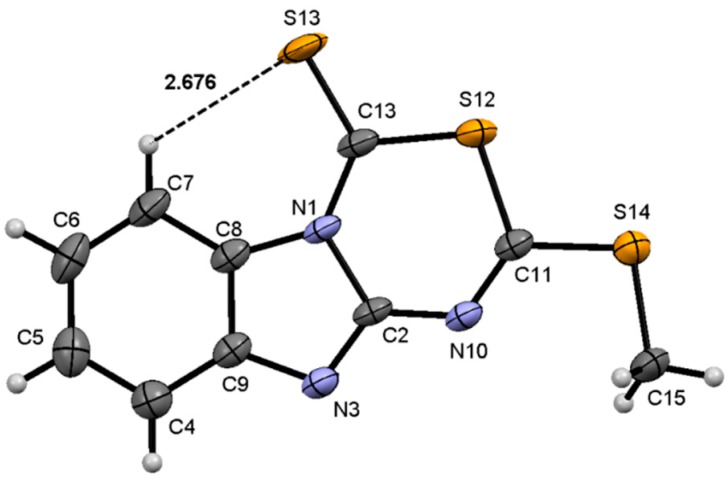
Molecular structure of compound **7**. Selected bond lengths (Å) and angles (°): S(12)–C(11) 1.760(2), S(12)–C(13) 1.744(2), S(13)–C(13) 1.629(2), S(14)–C(11) 1.739(2), S(14)–C(15) 1.796(3), N(3)–C(2) 1.301(3), N(3)–C(9) 1.394(3), N(1)–C(2) 1.433(3), N(1)–C(13) 1.372(3), N(10)–C(2) 1.355(3), N(10)–C(11) 1.286(4), C(11)–S(12)–C(13) 103.77(12), C(11)–S(14)–C(15) 101.18(13), C(2)–N(1)–C(13) 126.11(19), C(2)–N(10)–C(11) 121.6(2), N(1)–C(2)–N(3) 112.31(18), S(12)–C(11)–N(10) 126.27(16), N(10)–C(2)–N(1)–C(13) 2.4(4), N(1)–C(2)–N(10)–C(11) −4.1(3), C(7)–C(8)–N(1)–C(13) −3.0(4), N(10)–C(11)–S(12)–C(13) 2.0(2), N(10)–C(11)–S(14)–C(15)–6.1(2).

Analysis of the X-ray diffraction structure of compound **7** (Figure 4) shows the new fusioned heterocycle in the same plane of the benzimidazole ring. The values of the torsion angles N(10)–C(11)–S(12)–C(13) of 2.0(2)° and N(10)–C(2)–N(1)–C(13) of 2.4(4)°, are representative of this condition. These molecular arrangement, explains the interaction of the phenyl hydrogen with the sulfur atom whose geometric parameters are: H(7)∙∙∙S(13) of 2.68 Å and C(7)–H(7)∙∙∙S(13) of 121°; this interaction is responsible for the high frequency shift of the phenyl H7 observed in the ^1^H NMR spectrum. The bond distances N(3)―C(2) of 1.301(3) Å and N(10)–C(2) of 1.355(3) Å are of intermediate value for single and double bond character, whereas the N(1)–C(2) and N(10)–C(11) distances of 1.433(3) Å and 1.286(4) Å, are characteristics for a single and a double bonds, respectively. The bond distances and angles are in agreement with the proposed structure. 

**Figure 5:**
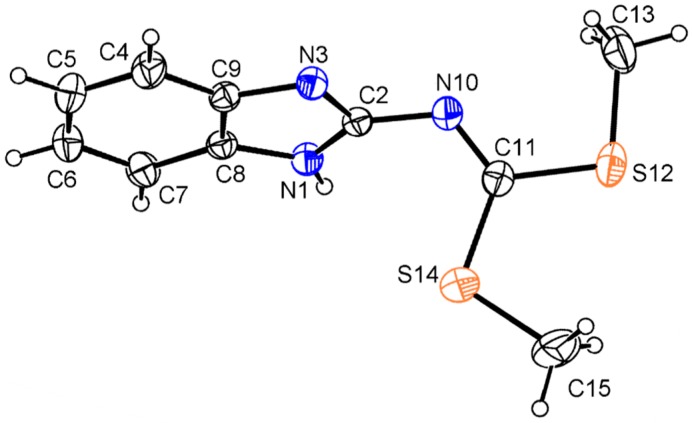
Molecular structure of compound **13**. Selected bond lengths (Å) and angles (°): S(12)–C(11) 1.745(2), S(12)–C(13) 1.786(3), S(14)–C(11) 1.748(2), S(14)–C(15) 1.795(3), N(1)–C(2) 1.338(3), N(1)–C(8) 1.383(3), N(3)–C(2) 1.335(3), N(3)–C(9) 1.388(3), N(10)–C(2) 1.383(3), N(10)–C(11) 1.273(3), C(11)–S(12)–C(13) 101.10(14), C(11)–S(14)–C(15) 104.73(16), C(2)–N(10)–C(11) 121.95(18), N(1)–C(2)–N(3) 113.69(17), N(1)–C(2)–N(10) 125.16(19), N(3)–C(2)–N(10) 120.78(19), S(12)–C(11)–S(14) 117.10(15), S(12)–C(11)–N(10) 120.23(17), S(14)–C(11)–N(10) 122.67(17), N(1)–C(2)–N(10)–C(11) −71.3(3), N(3)–C(2)–N(10)–C(11) 116.2(2), N(10)–C(11)–S(12)–C(13) −1.0(2), S(12)–C(11)–S(14)–C(15) 7.4(2), N(10)–C(11)–S(14)–C(15) −173.0(2), S(12)–C(11)–N(10)–C(2) 179.93(18), S(14)–C(11)–N(10)–C(2) 0.4(3).

The molecular structure of compound **13** is depicted in Figure 5. Benzimidazole NH prefers to form intermolecular N–H∙∙∙N, instead of intramolecular hydrogen bonding interactions to give a polymeric supramolecular structure. Thus the N=C(SMe)_2_ moiety is free for rotation, being located out of the mean benzimidazole ring plane as shown by the values of the torsion angles of −71.3(3)° for N(1)–C(2)–N(10)–C(11) and 116.2(2)° for N(3)–C(2)–N(10)–C(11). This geometric feature contrasts with the planar structure observed for the analogous derivatives of 2-aminobenzothiazole and 2-amino-1-methyl benzimidazole [16]. The N(1)–(2), N(3)–(2) and N(10)–(2) distance values of 1.338(3), 1.335(3) and 1.383(3) Å, are intermediate between single and a double bond character, compared with N(10)–C(11) of 1.273(3) Å, which has a double bond character.

**Figure 6:**
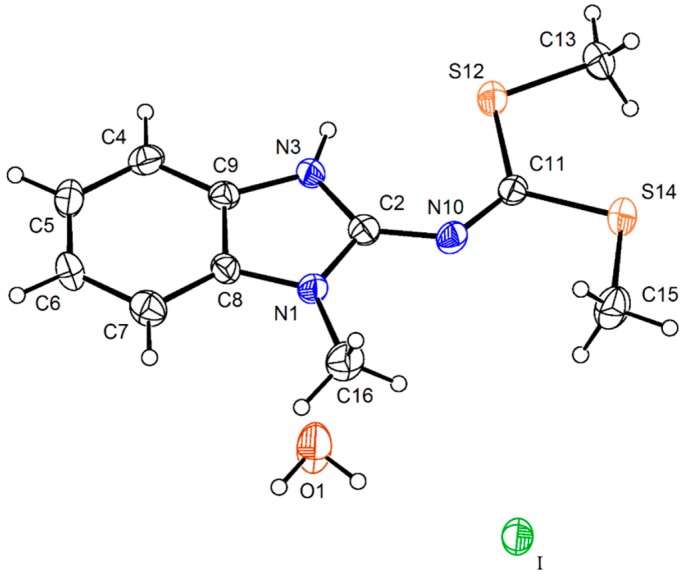
Molecular structure of the iodide salt **14** at 30% of probability. Selected bond lengths (Å) and angles (°): S(12)–C(11) 1.743(9), S(12)–C(13) 1.791(8), S(14)–C(11) 1.731(8), S(14)–C(15) 1.794(10), N(1)–C(2) 1.356(10), N(1)–C(8) 1.378(9), N(1)–C(16) 1.458(12), N(3)–C(2) 1.345(10), N(3)–C(9) 1.382(9), N(10)–C(2) 1.342(10), N(10)–C(11) 1.289(10), C(4)–C(5) 1.391(11), C(2)–N(1)–C(16) 125.3(6), C(2)–N(10)–C(11) 129.2(7), N(1)–C(2)–N(3) 107.9(6), N(1)–C(2)–N(10) 118.4(7), N(3)–C(2)–N(10) 133.8(7), S(12)–C(11)–S(14) 116.9(4), S(12)–C(11)–N(10) 123.7(6), S(14)–C(11)–N(10) 119.5(6), N(1)–C(2)–N(10)–C(11) 179.8(7), N(3)–C(2)–N(10)–C(11) −0.8(14), N(10)–C(11)–S(12)–C(13) −177.8(7), S(12)–C(11)–S(14)–C(15) 176.5(5), N(10)–C(11)–S(14)–C(15) −3.5(7), S(12)–C(11)–N(10)–C(2) 1.5(11), S(14)–C(11)–N(10)–(2) −178.5(6).

Compound **14** crystalized with one molecule of water (Figure 6). The intramolecular N(3)–H(3)∙∙∙S(12) hydrogen bonding interaction gives shape to a six membered ring with a N(3)∙∙∙S(12) distance of 3.081(6) Å and N(3)–H(3)∙∙∙S(12) angle of 120° which forces the planarity of the molecule. The angles N(1)–C(2)–N(10)–C(11) of 179.8(7)° and N(3)–C(2)–N(10)–C(11) of −0.8(14)° confirm the planarity of the N=C(SMe)_2_ an the value of N(10)–C(2) bond length of 1.342(10) Å, the strengthening of this bond. There is an intermolecular O1∙∙∙S12 interaction of 3.302(9) Å, instead of the S∙∙∙S interaction observed in the crystal structure of the neutral compound **15** [15].

## 3. Experimental Section

### 3.1. General Procedures

Melting points were measured on an Electrothermal IA apparatus and are uncorrected. IR spectra were recorded in a film on ZnSe using a Perkin-Elmer 16F PC IR spectrophotometer. ^1^H- and ^13^C-NMR spectra were recorded on a Varian Mercury 300 MHz (^1^H, 300.08; ^13^C, 75.46 MHz instrument). The spectra were measured with tetramethylsilane as internal reference following standard techniques. Physicochemical data is listed in Table 3. Crystallographic data (excluding structure factors) for the structures in this paper has been deposited in the Cambridge Crystallographic Data Centre as supplementary publication numbers CCDC **7** (1002932), **13** (1002930) and **14** (1002929). A summary of collection and refinement of the X-ray data is listed in Table 4. H atoms were treated as riding atoms, with C-H distances in the range of 0.93–0.96 Å and N-H distances of 0.82 Å. X-ray diffraction cell refinement and data collection: a Bruker SMART APEX Diffractometer and SAINT [17]. The SHELXS-97 programs were used to solve the structures [18]. PLATON [19] and *WinGX* [20] software was used to prepare material for publication. 2-Aminobenzimidazole (**3**) was a commercial product.

**Table 3:** Complementary data of compounds **6**, **7**, **9**–**14**, **16**, **17**.

Comp.	Yield (%)	Physical Appearance	M.p. (°C)	(cm^−1^)	*m/z* (%M^+^)	Elemental AnalysisFound (Calculated)		
						C	H	N
**6**	95	white crystals	263–264	3273, 1647		37.73 (37.40)	4.33 (4.18)	13.96 (14.54)
**7**	40	yellow crystals	134–135	1607, 1542	265(70)	46.30 (45.26)	2.72 (2.66)	15.20 (15.83)
**9**	80	yellow powder	198–199	3273, 3206, 1647	237+HI(61)	32.95 (32.87)	3.39 (3.28)	11.29 (11.50)
**10**	90	yellow powder	66–67		237(61)	49.93 (50.60)	4.78 (4.67)	17.29 (17.70)
**11**	92	yellow powder	148–150	3276, 3114 1646		34.25 (34.83)	3.79 (3.69)	10.66 (11.08)
**12**	86	yellow powder	85–86			53.05 (52.58)	5.12 (5.18)	16.15 (16.73)
**13**	25	white crystals	179–180	3469, 1624 1554	237(30)	50.51 (50.63)	4.76 (4.64)	17.28 (17.72)
**14**	90	white crystals				46.82 (34.83)	4.77 (3.69)	19.48 (11.08)
**16**	42	white powder	198–200					
**17**	40	white crystals	215–216	3129, 1616, 1573, 1543		48.25 (48.40)	4.07 (4.06)	18.37 (18.82)

**Table 4:** X-ray crystal data of compounds **7**, **13** and **14**.

Compound	7	13	14
**Unit Cell Information**			
Cell axes [Å] a	15.8405(4)	10.0640(20)	7.4895(7)
B	4.5179(1)	8.9376(18)	10.3023(9)
C	18.8892(6)	13.4110(30)	11.2194(10)
Cell angles [deg] α	90.00	90.00	110.270(1)
Β	126.546(2)	107.480(30)	95.492(2)
Γ	90.00	90.00	104.655(1)
Crystal system	Monoclinic	Monoclinic	Triclinic
Space group	P 2_1_/c	P 2_1_/c	P-1
Molecular Formula	C_10_H_7_N_3_S_3_	C_10_H_11_N_3_S_2_	C_11_H_16_N_3_OS_2_I
Density [g cm^−1^]	1.62	1.37	1.71
Formula weight	265.4	237.3	397.3
No. Form. Units Z	4	4	2
**Reflection Data**			
No. Meas.	10175	10627	7445
No. Uniq.	2173	2023	2706
No. Obs.	1971	1896	2301
**Current Refinement**			
No. Refln.	2173	2023	2706
No. Param.	147	136	166
Delta-rho [eÅ^−3^] max, min	0.361, −0.518	0.392, −0.332	1.012, −1.134
R_all, R_obs	0.045, 0.042	0.045, 0.043	0.079, 0.065
wR2_all, wR2_obs	0.117, 0.113	0.122, 0.120	0.130, 0.125

### 3.2. Procedures to Obtain 2-Aminobenzimidazole Sulfur Derivatives

#### 3.2.1. 1,3-Dimethyl-1,3-dihydrobenzimidazol-2-ylideneammonium Iodide (**6**)

In a 100 mL round-bottom flask, a solution of sodium hydroxide (0.3 g, 7.52 mmol) in water (0.5 mL), and a solution of 2-aminobenzimidazole (**3**, 1.0 g, 7.52 mmol) in DMF (6 mL) were successively added. The mixture was cooled on an ice bath and stirring for 30 min, then methyl iodide (0.94 mL, 15.0 mmol) was added and stirring was continued for 24 h. The reaction was quenched by the addition of cold water (100 mL). The precipitated solid was filtered off, washed with water and recrystallized from ethanol. After air drying, 1.95 g (90%) of white crystals were obtained. ^1^H-NMR [δ, ppm, DMSO-*d*_6_]: 8.72 (b, 2H, ^+^NH_2_), 7.55 (m, 2H, Ar) and 7.35 (m, 2H, Ar) 3.67 (s, 6H, N-CH_3_) ^13^C-NMR [δ, ppm, DMSO-*d*_6_]: 150.73 (s, C2), 130.68 (s, C8, C9), 124.05 (s, C5, C6), 110.81(s, C4, C7).

#### 3.2.2. 2-Methylthio-4*H*-[1,3,5]thiadiazino[3,4-a]benzimidazole-4-thione (**7**)

In a 100 mL round-bottom flask, a solution of sodium hydroxide (0.4 g, 10 mmol) in water (0.5 mL), and a solution of 2-aminobenzimidazole (**3**, 1.33 g, 10 mmol) in DMF (6 mL) were successively added. The mixture was cooled on an ice-water bath and stirring for 30 min. Then, the following reactants were successively added: (a) carbon disulfide (0.6 mL, 10 mmol); (b) sodium hydroxide aqueous solution 20 M (0.5 mL 10 mmol); (c) carbon disulfide (0.6 mL, 10 mmol) and (d) methyl iodide (1.25 mL, 20 mmol). Portionwise addition and a delay of 30 min between additions were necessary in order to complete the reaction. Stirring was continued for 24 h and cold water (100 mL) was added to the mixture. The precipitated solid was filtered off, washed with water, and purified by recrystallization from chloroform. After air drying, 1.06 g (40%) of yellow crystals were obtained.

#### 3.2.3. 2-Aminobenzimidazole-1-carbodithioic Acid Methyl Ester (**8**)

The same procedure and quantities as described for **7** were used following the sequence: (a) carbon disulfide (0.6 mL, 10 mmol); (b) methyl iodide (0.63 mL, 10 mmol). Compound **8** was purified by recrystallization from ethanol to obtain 1.47 g (66%) of yellow crystals.

#### 3.2.4. 1-Methyl-3-(methylthiocarbonothioyl)-1*H*-benzo[*d*]imidazol-2(3*H*)-iminium Iodide (**9**)

The same procedure as described for **8** was used following the sequence: (a) carbon disulfide (0.6 mL, 10 mmol); (b) methyl iodide (1.25 mL, 20 mmol). Compound **9** was purified by recrystallization from ethanol to obtain 2.41 g (66%) of yellow powder.

#### 3.2.5. 2-Imino-3-methyl-2,3-dihydrobenzimidazol-1-carbodithioic Acid Methyl Ester (**10**)

The same procedure as described for **9** was used, adding (c) sodium hydroxide aqueous solution (0.5 mL, 10 mmol). After standing, 1.42 g (60%) of compound **10** precipitated as a yellow powder.

#### 3.2.6. *N*-(1-Methyl-3-(methylthiocarbonothioyl)-1*H*-benzo[*d*]imidazol-2(3H)-ylidene)methanaminium Iodide (**11**)

In 100 mL round-bottom flask a solution of compound **10** (1.0 g, 4.2 mmol) in DMF (6 mL) and methyl iodide (0.26 mL, 4.2 mmol) were successively added. The mixture was stirred for 24 h at room temperature and cold water (100 mL) was then added to the mixture. The precipitated solid was filtered off and washed with water. After air drying, 1.08 g of yellow powder (67.5%) were obtained.

#### 3.2.7. (*E*)-3-Methyl-2-methylimino-2,3-dihydrobenzimidazole-1-carbodithioic Acid Methyl Ester (**12**)

Compound **11** (1.0 g, 2.63 mmol) was reacted with aqueous sodium hydroxide solution (5.3 mL, 0.5 M). The resulting precipitate was washed with enough water to give 0.48 g (72.7%) of a yellow powder.

#### 3.2.8. Dimethyl 1*H*-benzo[*d*]imidazol-2-ylcarbonimidodithioate (**13**)

In 500 mL round-bottom flask, a solution of sodium hydroxide (2.0 g, 50 mmol) in water (2.5 mL), and a solution of 2-aminobenzimidazole **3** (6.65 g, 50 mmol) in DMF (30 mL) were successively added. The mixture was cooled on an ice-water bath, stirred for 30 min and carbon disulfide (3.0 mL, 50 mmol) was added. The mixture was refluxed for 4 h, the solution was cooled on an ice bath and the following reactants were successively added: (a) sodium hydroxide aqueous solution 20 M (2.5 mL, 50 mmol); (b) methyl iodide (6.25 mL, 100 mmol). Portionwise addition and a delay of 30 min between additions were necessary in order to complete the reaction. Stirring was continued for 24 h and cold water (500 mL) was added to the mixture. The precipitated solid was filtered off, washed with water and purified by recrystallization in ethanol to obtain 2.96 g (25%) of white crystals.

#### 3.2.9. 2-(*Bis*(methyltio)methyleneamino)-1-methyl-1*H*-benzo[*d*]imidazol-3-ium Iodide (**14**)

In 100 mL round-bottom flask a solution of compound **13** (1.0 g, 4.2 mmol) in of DMF (6 mL) and methyl iodide (0.3 mL, 4.8 mmol) were added, The mixture was cooled on an ice-water bath and stirring for 12 h and cold water (100 mL) was added to the mixture. The precipitated solid was filtered off, washed with water and purified by recrystallization in ethanol to obtain 1.32 g (85.7%) of white crystals.

#### 3.2.10. Dimethyl 1-methyl-1*H*-benzo[*d*]imidazol-2-ylcarbonodithioimidate (**15**)

Compound **14** (1.32 g, 3.48 mmol) was reacted with sodium hydroxide aqueous solution (7.2 mL, of 0.5 M). The resulting precipitate was washed with enough water to give 0.73 g (85%) of white crystals.

#### 3.2.11. (1,3-Dihydrobenzimidazol-2-ylidene)-dithiocarbamic Acid methyl Ester (**16**) and 2-(*bis*(methyl- thio)methyleneamino)-1*H*-benzo[*d*]imidazol-3-ium Iodide (**17**)

The same procedure and quantities as described for **13** were used, but half the amount of methyl iodide (3.13 mL, 50 mmol) was used. The precipitated solid was dissolved in ethanol and 1.2 g of compound **17** precipitated as white crystals. From the ethanol solution, 0.8 g of compound **16** precipitated as a white powder.

## 4. Conclusions

We have demonstrated that by a careful control of the stoichiometric quantities and addition sequences as well as the temperature, the reactions of 2-aminobenzimidazole with NaOH, CS_2_ and CH_3_I allow the selective functionalization of the benzimidazole ring with *N*-dithiocarbamate, *S*-methyldithiocarbamate or dimethyldithiocarboimidate groups. The imidazolic hydrogen atom is more acid than that of the amino group in 2-aminobencimidazole, thus both endocyclic nitrogen atoms are methylated with methyl iodide in neutral or basic media. In the reaction with CS_2_ at ice-water bath (4 °C) temperature, the endocyclic dithiocarbamate is formed when the first molar equivalent of CS_2_ is added whereas the exocyclic dithiocarbamate is formed after the second molar equivalent of CS_2_ is added. The regiochemistry of this reaction is shifted to form the exocyclic dithiocarboimidate as the only product when the reaction is performed in refluxing DMF. The dimethyldithiocarboimidate group of compound **13** is out of the benzimidazole plane, in contrast to the planar structure of the analogous compounds **15** and **2** derived from 2-amino-1-methylbenzimidazole and 2-aminobenzothiazole, respectively. The preferred conformers of iminium salts, compounds **9** and **11**, and the corresponding neutral compounds, **10** and **12**, were determined. Compound *(Z)*-**11** was observed in CDCl_3_, whereas a *Z*-*E* isomerization occurred in DMSO-*d*_6_. Compound *(E)*-**11** is selectively deprotonated to form the free base *(E)*-**12**. 
